# Spin glass–like transition in the structurally disordered intermetallic compound Sm_2_Au_1.1_Ge_2.9_

**DOI:** 10.1038/s41598-025-29384-6

**Published:** 2025-11-23

**Authors:** L. S. Litzbarski, M. J. Winiarski, T. Klimczuk, K. Synoradzki, M. Matczak, P. Skokowski, A. Bajorek, B. Andrzejewski

**Affiliations:** 1 Faculty of Applied Physics and Mathematics, Gdansk University of Technology, Narutowicza 11/12, 80-233 Gdansk, Poland; 2 Advanced Materials Center, Gdansk University of Technology, Narutowicza 11/12, 80-233 Gdansk, Poland; 3 Faculty of Electrical and Control Engineering, Gdansk University of Technology, Narutowicza 11/12, 80-233 Gdansk, Poland; 4 Institute of Molecular Physics, Polish Academy of Science, Smoluchowskiego 17, 60-179 Poznan, Poland; 5 A. Chełkowski Institute of Physics, University of Silesia in Katowice, 75 Pułku Piechoty 1, 41-500 Chorzów, Poland

**Keywords:** Sm_2_AuGe_3_, Spin glass, Magnetic memory effect, Time relaxation, Disordered AlB_2_- type structure, Chemistry, Materials science, Physics

## Abstract

The novel intermetallic compound Sm_2_Au_1.1_Ge_2.9_ was synthesized using an arc-melting method. A detailed analysis of its crystallographic structure, magnetic and thermal properties has been documented. Powder X-ray diffraction analysis indicated that this compound crystallizes in a disordered variant of the AlB_2_-type structure (space group *P*6/*mmm*, no. 191) with lattice parameters *a* = 4.2495(1) Å and *c* = 4.1300(1) Å. The exact nature of magnetic properties of this intermetallic compound was investigated both by AC and DC magnetization measurements including time evolution of remnant magnetization and magnetic memory effect studies. The investigation of magnetic properties was supplemented by measurements of heat capacity with and without an applied magnetic field. The new Sm_2_Au_1.1_Ge_2.9_ compound is found to exhibit a cluster glass transition at surprisingly high temperature *T*_f_(39 Hz) = 21.9 K. Such a high phase transition temperature makes this material a promising candidate for technical applications e.g. in magnetic memory devices.

## Introduction

Spin glasses are defined as materials that exhibit random magnetism as a result of the coexistence of competing antiferromagnetic and ferromagnetic interactions^1^. This group of materials has been discovered during studies of diluted magnetic alloys such as Au:Fe3% owing to an employment of the AC susceptibility measurement technique^2^. Thes quickly became an object of interest of solid-state physics scientists due to their unusual properties e.g. a magnetic memory effect and frequency dependent shift of AC magnetization. These features make spin glasses promising candidates for innovative electronic devices, including systems to encode information^3^. On the other hand, spin glasses are one of the simplest types of disordered materials, which makes them a valued object of metastability studies in glassy systems. Nowadays, diluted magnetic alloys, which reveal glassy behavior are often referred to as *canonical spin glasses* in order to distinguish them from reported subsequently intermetallic compounds exhibiting spin glass-like features. Alongside *canonical spin glasses*, there are many groups of materials similar to spin glasses, one of them being *cluster glasses,* in which magnetic moments occur in clusters rather than as individual spins^4^.

In general, spin glass like materials exhibit slow relaxation of magnetization, and multiple degenerated ground state meaning they can retain information about their magnetic state for long periods. This property can be leveraged in high-density data storage devices. Spin glass devices are expected to be part of neural networks as content-addressable memories (CAMs) or associative memory devices. These memory devices should be capable of learning and recognizing previously learned patterns, should be robust and noise-tolerant. Due to the multiple degenerate energy states, they should also store large amounts of data. Moreover, these materials may serve in applications requiring protection from unwanted magnetic fields, such as in sensitive electronic instruments that need to be shielded from interference^2^.

**Fig. 1:**
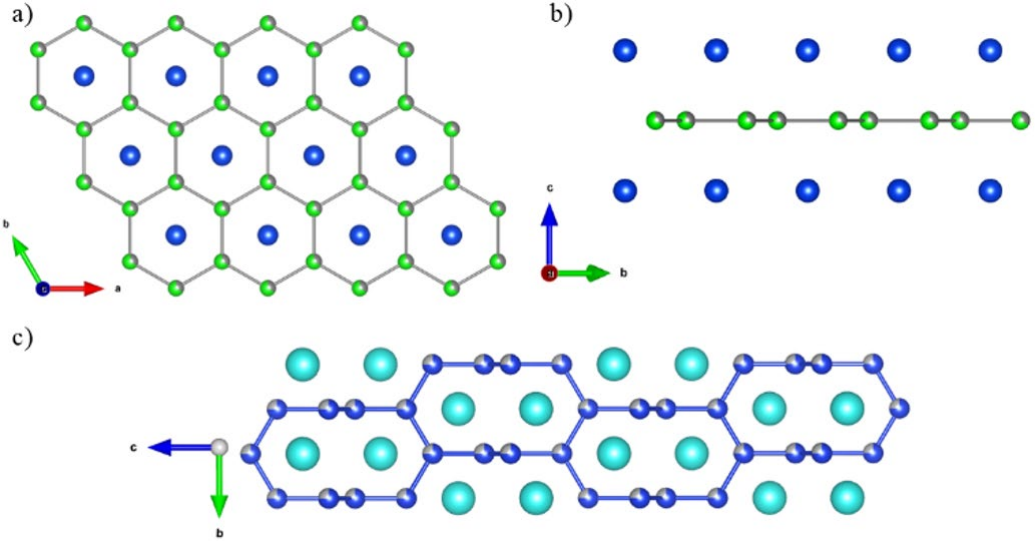
Crystal structure of: (**a**, **b**) Sm_2_Au_1.1_Ge_2.9_ (AlB_2_-type)– big blue spheres are samarium atoms, smaller ones represent gold and germanium, which stochastically occupy hexagonal site with ratio close to 1:3; (**c**) Gd_2_AgSi_3_ (α-ThSi_2_ type) – large spheres represents gadolinium atoms, while the smaller one stand for silver and silicon randomly distributed with probability 1:3 (all drawings were made employing VESTA software^22^).

Rare earth (*RE*) intermetallic compounds with a nominal stoichiometry *RE*_2_*TrX*_3_ (*Tr* – transition metal, *X*—semimetal) are known to present diverse physical properties. For example, La_2_NiGe_3_^5^ and other analogues with nonmagnetic *RE* elements^6,7^ are usually superconductors, while compounds containing *f*-block elements are predicted to exhibit intriguing magnetic properties. A complex magnetic behavior was observed in *RE*_2_AgGe_3_ compounds^8^ or in Gd_2_AgSi_3_^9^ and Pr_2_NiGe_3_^10^ with a double magnetic transition, while *RE*_2_NiSi_3_ intermetallics tend to display a large magnetocaloric effect^11–13^. Recently published *RE*_2_PdGe_3_ and *RE*_2_PtGe_3_ series^14–19^ reveal a glass-like spinning behavior caused by the coexistence of magnetic frustration and structural disorder. In general, *RE*_2_*TrX*_3_ compounds crystalize in AlB_2_ derived crystal structures, which are commonly represented by a hexagonal structure (space group *P*6/*mmm*, no 191) or a tetragonal α-ThSi_2_-type one (space group *I*4_1_/*amd*, no. 141). Ternary *RE*_2_*TrX*_3_ compounds crystallizing in the hexagonal structure of the AlB_2_ type (Fig. 1a, b) are composed of parallel layers of *RE* ions, which determine their magnetic moments^20^. These layers are separated by graphite-like nets formed of *Tr* and *X* atoms, which are randomly distributed with probability 1:3. This structural disorder causes a variation of the local environment around *RE* ions, which may lead to the existence of magnetic frustration. Additionally, comparable values of lattice parameters *a* and *c* increase random exchange interactions between *RE* ions, which favor the formation of magnetic clusters^11^. Moreover, it can be noted that the replacement of La^3+^ ion with other lanthanides cause a shrinkage of the unit cell and generate strains in the crystal structure^19^. This behavior is known as a chemical pressure effect and results from the lanthanide contraction. In case of α-ThSi_2_ type structure Th positions are occupied by *RE* ions, while Si are replaced by a quarter of *Tr* atoms and three quarters of *X* atoms. This crystal structure can be viewed as a “twisted” AlB_2_, which is shown in Fig. 1c. The relationship between different structures of the AlB_2_ aristotype can be described using the Bärnighausen tree^21^.

In this article, we report the successful synthesis of a new intermetallic compound Sm_2_Au_1.1_Ge_2.9_, which is one of the first member of the *RE*_2_AuGe_3_ series, next to compound with Yb^23^ and Ce, to crystalize in a hexagonal structure^24^. The magnetic properties investigations established that the studied compound exhibits a cluster glass transition above *T* = 20 K, which makes it a promising candidate for applications in technology e.g. as magnetic devices components.

## Experimental

The polycrystalline sample of Sm_2_Au_1.1_Ge_2.9_ was synthetized under an inert (Zr—gettered pure Ar) atmosphere using an arc melting technique. Constituent amounts of a high purity chemical elements (> 99.99%) was weighted considering 10% molar excess of Sm due to a significant volatility of this metal and then melted together in an arc-furnace MAM-1 GmbH Edmund Bühler. The ingot was rotated several times and remelted to improve the homogeneity of the synthetized material. The sample was weighed after the melting process, and the observed mass losses were comparable to the weight of the excess Sm used, which can suggest that the chemical composition of the ingot is close to the nominal stoichiometry. A sample with a total weight of ~ 0.5 g was synthesized. No thermal treatment was applied to the sample after synthesis.

The sample was characterized by powder X-ray diffraction (pXRD) using a Bruker D2Phaser diffractometer equipped with an XE-T detector (CuK_α_ radiation). The obtained pXRD pattern was analyzed by the Rietveld profile refinement using the GSAS-II package^25^.

Scanning electron microscopy (SEM) images and elemental compositions were acquired using an FEI Nova NanoSEM 650 equipped with an energy-dispersive spectroscopy (EDS) attachment. Imaging and excitation of the characteristic radiation of the elements were conducted at an electron energy of 30 keV. Licensed instrument software was used to analyze EDS data using the P/B ZAF (non-standard) method.

The sample density was determined using Archimedes’ method. The measurement was made using isopropyl alcohol [(CH_3_)_2_CHOH] and an electronic balance Radwag (Radom, Poland) XA 110.4Y.A.

The electronic structure analysis was performed using X-ray photoelectron spectroscopy (XPS) on a Physical Electronics (PHI 5700/660) spectrometer, which operated under ultra-high vacuum (10^−9^ Torr) conditions in a UHV cluster, employing a monochromatic Al Kα X-ray source (1486.6 eV). Initially, the sample was placed in a pre-chamber and maintained under vacuum at 10^−8^ Torr for a minimum of 12 h. It was then transferred to the preparation chamber and cleaved under UHV conditions (10^−9^ Torr) to achieve a fresh surface. The cleaved sample was subsequently moved immediately to the measurement chamber for analysis. Survey spectra were collected at a transition energy of 187.85 eV and core level lines were measured at a transition energy of 23.5 eV. The acquired spectra were processed using XPSPEAK41 software, and the deconvolution of the core level lines was carried out using the Shirley background^26^ method along with the Gaussian–Lorentzian line shape.

Physical properties of Sm_2_Au_1.1_Ge_2.9_ were investigated by specific heat and magnetization measurements performed on a Quantum Design Physical Property Measurement System (PPMS). A vibrating sample magnetometer (VSM) option was used to perform DC magnetic measurements for different applied magnetic field (up to 9 T) both in Zero Field Cooling mode (ZFC) as well as in Field Cooling mode (FC). The AC magnetization data were collected employing the AC- Measurement System (ACMS) in the frequency range 39 Hz—10 kHz with the magnetic field amplitude μ_0_*H*_ac_ = 5 mT. Heat capacity investigations were carried out with and without an applied magnetic field by a standard thermal relaxation technique in the temperature range 1.9 K < *T* < 300 K.

## Results and discussion

Similar to reported earlier *RE*_2_*TrX*_3_ compounds e.g. Tb_2_Pd_1.25_Ge_2.75_^14^, Tm_2_Ni_0.93_Si_2.93_^12^ and Dy_2_Pt_1.15_Ge_2.85_^18^, the fully stoichiometric Sm_2_AuGe_3_ reveals the significant presence of an additional phase, which cannot be removed by a thermal annealing process. In order to obtain a single phase sample, it was necessary to synthetize the intermetallic compound with a defective crystal structure by deliberately changing Au:Ge ratio. The influence of adjusting composition of graphite-like layers on *RE*_2_*TrX*_3_ structure stability was widely discussed in our previous articles^27,28^ The best result was achieved for the stoichiometry Sm_2_Au_1.1_Ge_2.9_.

### Crystal-chemical characterization

**Fig. 2:**
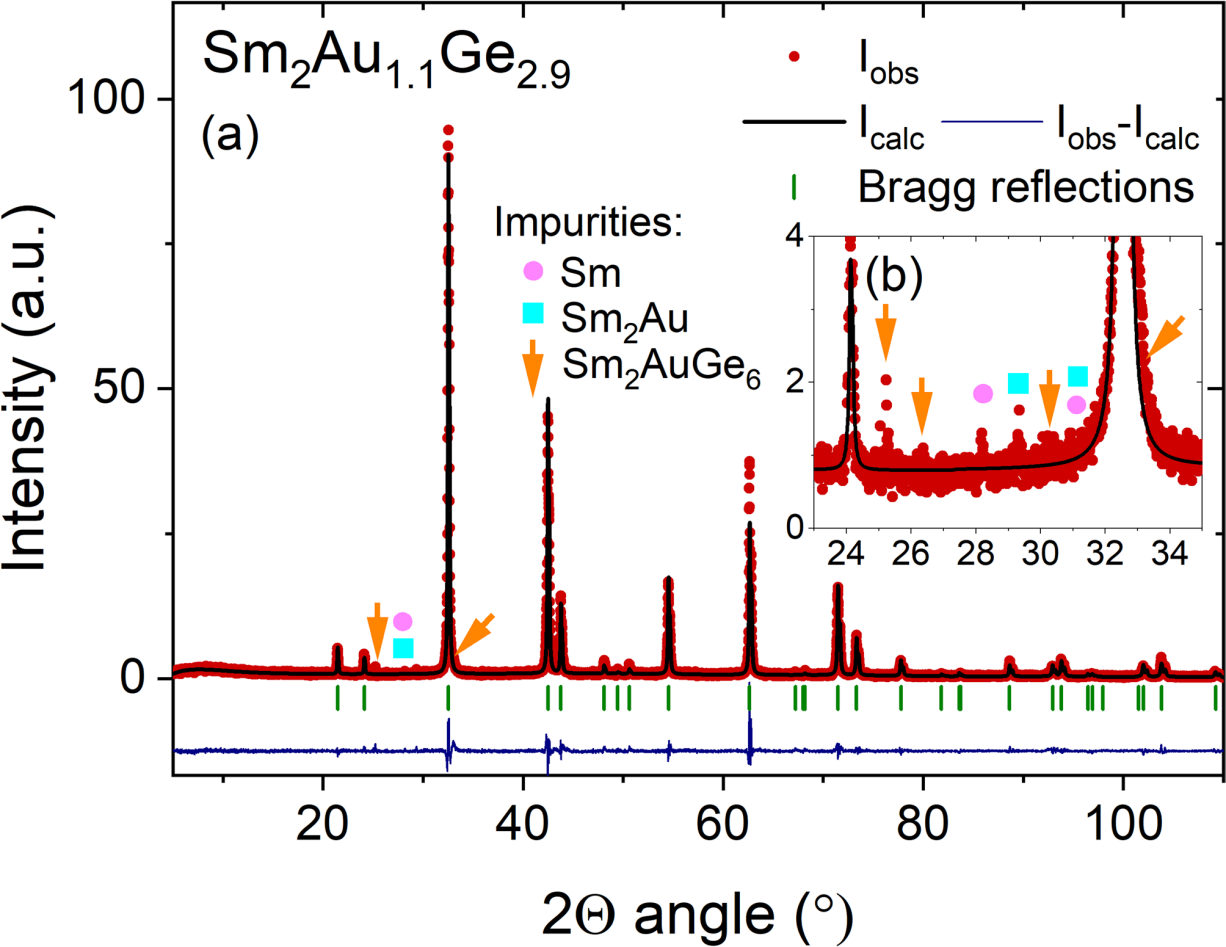
Rietveld refinement of powder XRD data for Sm_2_Au_1.1_Ge_2.9._ Observed data and calculated intensity are represented by red circles and black line respectively. The difference is shown in the lower part by solid blue line. Green vertical ticks correspond to Bragg peaks for space group *P*6/*mmm* (no. 191). Inset shows enlarged pXRD patterns in the range 2*θ* = 28°—34°. Pink circles indicate reflections from Sm, blue squares from Sm_2_Au, and orange arrows correspond to Sm_2_AuGe_6_.

In Fig. 2 the pXRD patterns for Sm_2_Au_1.1_Ge_2.9_ are shown, which can be well indexed by AlB_2_ derived structure with space group *P*6/*mmm*. In addition to the peaks associated with the main phase, we also observed peaks originating from trace amounts of the impurity phases identified as Sm (0.1165(7) wt. %), Sm_2_Au (0.415(2) wt. %) and Sm_2_AuGe_6_ (1.9(2) wt. %). More details are presented in Fig. 2b. However, based on the low intensity of the reflections associated with these phases, it can be concluded that they are present only as minor constituents. It should be noted that, unlike in many other *RE*_2_*TrX*_3_ compounds, the (*00 l*) reflection in Sm_2_Au_1.1_Ge_2.9_ is not significantly broadened compared to other reflections. This indicates the absence of pronounced anisotropic broadening or stacking faults in this material. Such broadening effects have previously been reported in related compounds such as Gd_2_Pt_1.1_Ge_2.9_^28^, Nd_2_PtGe_3_^15^ and Ce_2_PdGe_3_^29^. The calculated values of lattice parameters are equal *a* = 4.2495(1) Å and *c* = 4.1300(1) Å (more crystallographic data are gathered in Table 1) implying that a *c/a* ratio is about 1. Sm_2_Au_1.1_Ge_2.9_ crystallizes in a disordered variant of the AlB_2_-type structure as confirmed by a lack of superstructure reflections, which should be observed in case of an ordered variant represented by Ca_2_PdGe_3_^17^. The disordered *RE*_2_*TrX*_3_ structure has a random distribution of *Tr* and *X* atoms at honeycomb nets, which occur with probability 1:3. Furthermore, minor differences in lattice parameters cause that strengths of interactions between the nearest neighbors (NN) and the next-nearest neighbors (NNN) are comparable, which promote magnetic frustration^30^. The coexistence of structural disorder and magnetic frustration in Sm_2_Au_1.1_Ge_2.9_ establish favorable conditions for a spin glass-like state formation, which was investigated by physical properties measurements. In such a scenario, when the degree of disorder is moderate, finite-size regions with correlated spins can form, giving rise to a *cluster-glass* state rather than a conventional spin glass. Here, magnetic moments within each cluster are aligned ferromagnetically, but the inter-cluster interactions remain random and frustrated, resulting in collective freezing at low temperatures, as observed in Sm_2_Au_1.1_Ge_2.9_.

**Table 1:** Structural parameters determined for a new intermetallic compound Sm_2_Au_1.1_Ge_2.9_

Nominal composition	Sm_2_Au_1_Ge_3_
Optimal composition	Sm_2_Au_1.1_Ge_2.9_
Experimental composition	Sm_2.05(2)_Au_1.29(2)_Ge_2.66(3)_
Space group	*P*6*/mmm* (No. 191)
*a* (Å)	4.2495(1)
*c* (Å)	4.1300(1)
*V* (Å^3^)	64.589(2)
Molar weight (g mol^−3^)	728.04
Density (g cm^−3^)	9.198
Measured density (g cm^−3^)	9.17(6)
*RE* (1*a*)	x = y = z = 0;
	U_iso_ = 0.0277(4) Å^2^
Au (2*d*)	x = 1/3 y = 2/3 z = 0.5;
	U_iso_ = 0.0266(7) Å^2^
	Occ. = 0.275
Ge (2*d*)	x = 1/3 y = 2/3 z = 0.5;
	U_iso_ = 0.0266(7) Å^2^
	Occ. = 0.725
Figures of merit:	
R (%)	11.4
wR (%)	15.1
GOF	1.34

**Fig. 3:**
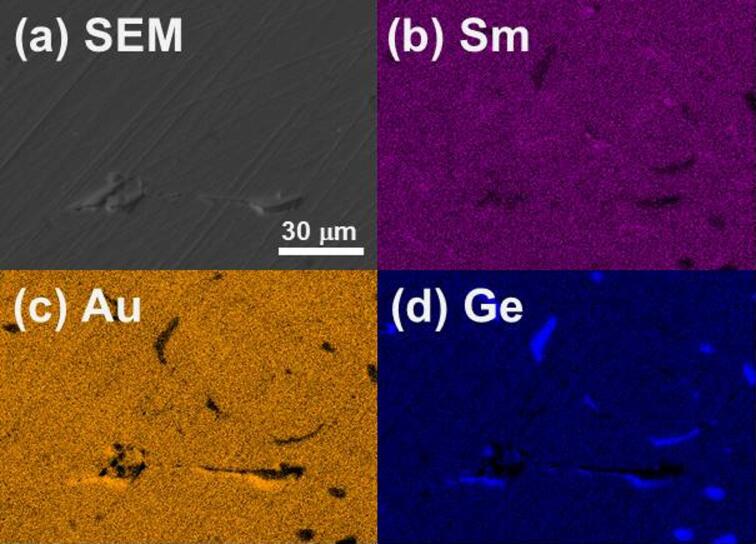
SEM image (**a**) is accompanied by respective EDS maps depicting individual elements of Sm (**b**), Au (**c**), and Ge (**d**) for Sm_2_Au_1.1_Ge_2.9._

The EDS technique was employed to measure the actual chemical composition of the resulting sample. The average chemical composition, determined by analyzing multiple locations on the surface (Fig. 3a), is Sm_2.05(2)_Au_1.29(2)_Ge_2.66(3)_. This value aligns with both the nominal composition Sm_2_AuGe_3_ and that derived from pXRD analysis Sm_2_Au_1.1_Ge_2.9_. EDS mapping showed that all elements are mostly uniformly distributed on the sample surface (Fig. 3b–d). However, we observed small Ge-rich precipitates (it can be Sm_2_AuGe_6_ phase identified in the pXRD pattern). Nonetheless, these are not expected to exert a substantial impact on the physical properties we assessed. EDS mapping revealed that all elements are predominantly uniformly distributed across the sample surface (Fig. 3b–d). Small Ge-rich precipitates were also detected, which may originate from Ge oxides—consistent with the signal observed in the XPS scans. Alternatively, they could be associated with the Sm_2_AuGe_6_ phase identified in the pXRD patterns. In either case, their presence is not expected to significantly influence the physical properties investigated.

**Fig. 4:**
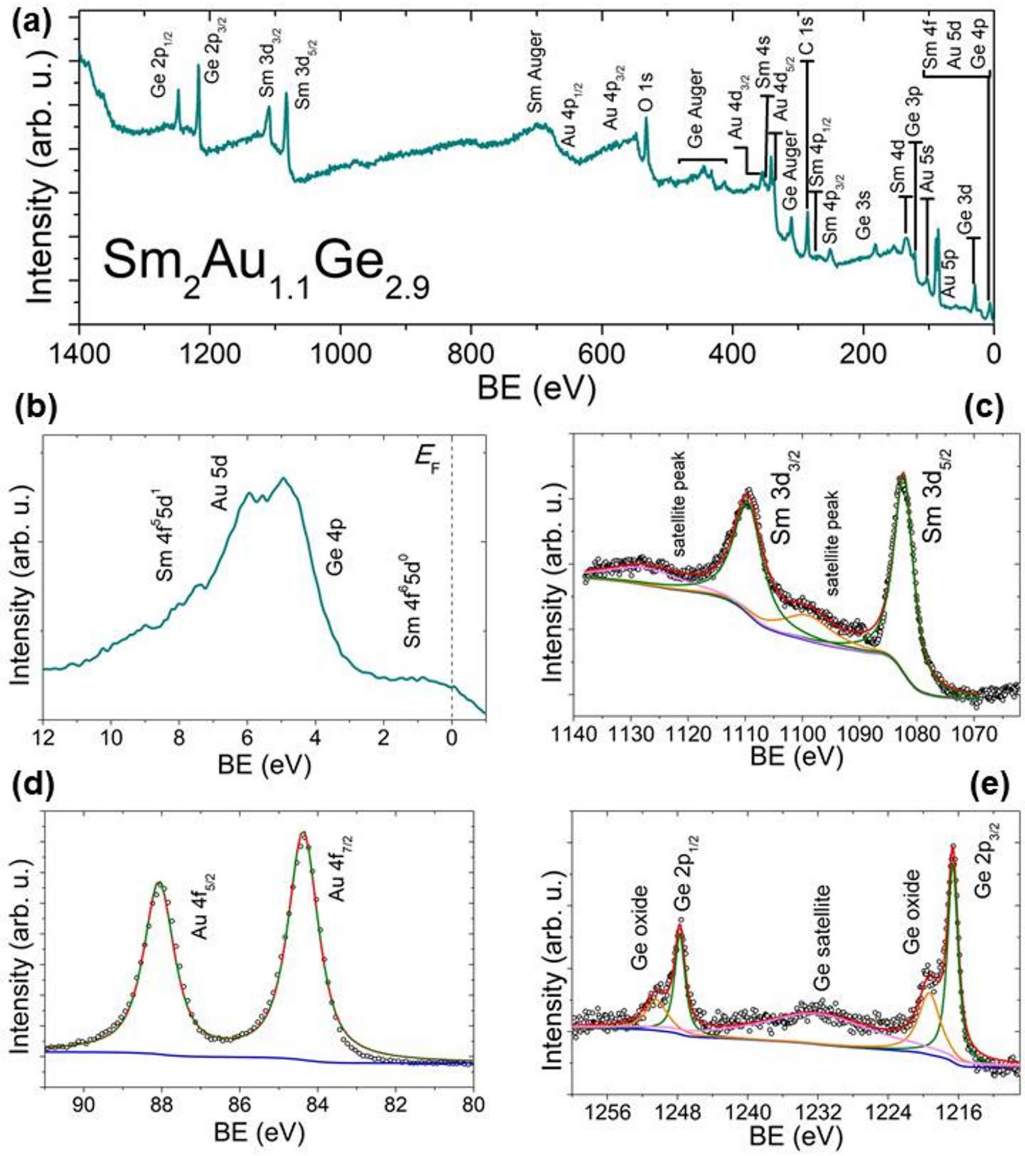
XPS spectra for Sm_2_Au_1.1_Ge_2.9_: (**a**) spectrum in a wide binding energy range. All core levels have been identified, (**b**) valence band spectrum, (**c**) Sm 3*d* spectrum, (**d**) Au 4f. spectrum, (**e**) Ge 2*p* spectrum.

Samarium (Sm), like cerium (Ce), europium (Eu) and ytterbium (Yb), can exist in two valence states, unlike other rare earth elements that typically only exist in the 3 + state. The valence state of a rare earth metal ion significantly influences the physical properties of the chemical compound in which it is found. In some cases, the valence of a rare earth ion may be mixed or fluctuating, leading to unusual behavior in various physical properties. One method to determine the valence state is X-ray photoelectron spectroscopy (XPS). Consequently, we conducted XPS measurements on a sample of the compound Sm_2_Au_1.1_Ge_2.9_ to ascertain the valence state of Sm in this compound. The results of the XPS measurements are collected in Fig. 4. XPS spectrum obtained over a wide range of binding energies (Fig. 4a) revealed peaks corresponding to the elements present in the Sm_2_Au_1.1_Ge_2.9_ compound, along with peaks from carbon and oxygen that stem from atmospheric components in the measurement chamber. All observed peaks were analyzed and described. Near the Fermi level *E*_F_, the XPS spectrum is characterized by a broad feature centered around 5 eV, which includes peaks from Sm 4f., Au 5*d*, and Ge 4*p* states. The absence of a distinct peak at *E*_F_ suggests that the predominant valence state of Sm is likely 3 +^31^. On the other hand, shoulder structure close to *E*_F_ may indicate a small contribution from Sm 2 + states. A similar spectral shape is also observed in other Sm-based compounds^32^.

The spectra for the Au 4f. states reveal two distinct peaks at binding energies of 84.4(1) eV and 88.1(1) eV. These peaks result from spin–orbit (SO) interaction, which in this case have a strength of of 3.7 eV. Figure 4d illustrates the deconvolution of the Au 4f. peaks. A similar pattern is observed in the Ge 2*p* states, where the spectrum primarily shows two peaks located at binding energies of 1216.7(1) eV and 1247.9(1) eV. The SO interaction in this instance is 31.2 eV. The spectrum also shows two distinct peaks at 1250.5(1) eV and 1219.4(1) eV, which are likely associated with Ge oxides. Additionally, a broad peak centered at 1232.4(1) eV related to Ge losses is observed between these two pair of peaks. The positions of the main Au and Ge peaks agree very well with literature values for the pure elements^33^ confirming that both elements are present predominantly in the Au(0) and Ge(0) states.

Similar for other Sm-based materials, XPS spectrum for 3*d* Sm states shows two peaks located at energies 1109 eV and 1082 eV formed by SO splitting of Sm^3+^ states (SO splitting is ~ 27 eV). The XPS spectrum does not show peaks attributable to Sm^2+^ states, which should appear below peaks derived from Sm^3+^ states. Based on the XPS measurements, it can be concluded that the valence state of Sm in the Sm_2_Au_1.1_Ge_2.9_ compound is 3 + . The XPS results obtained for Sm_2_Au_1.1_Ge_2.9_ are very similar to those obtained for the Ni sample (Sm_2_Ni_0.87_Si_2.87_), where the authors also observed peaks associated only with Sm^3+^ states^34^.

### Magnetic properties

**Fig. 5:**
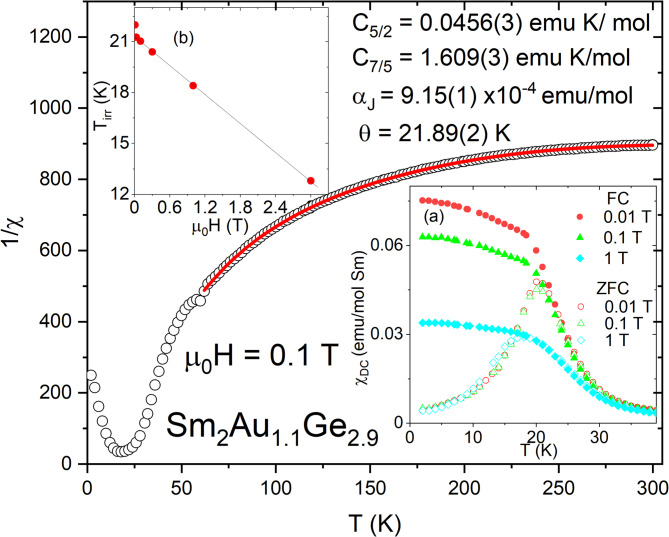
The temperature dependence of the inverse magnetic susceptibility for Sm_2_Au_1.1_Ge_2.9_. The red line is fit to modified Curie – Weiss law (see text for details). Inset (**a**): Temperature dependence of DC magnetic susceptibility (χ = *M*/*μ*_0_*H)* for different magnetic fields in zero-field-cooled (ZFC) and field-cooled (FC) mode. Inset (**b**): The dependence of the irreversibility temperature (*T*_*irr*_) for Sm_2_Au_1.1_Ge_2.9_ as a function of applied field μ_0_H.

In order to verify the hypothesis about Sm valence state DC magnetization, measurements were carried out. Figure 5 presents the inverse of molar magnetic susceptibility (1/*χ* = *μ*_0_*H/M*) for Sm_2_Au_1.1_Ge_2.9_ as a function of temperature in a range *T* = 2—300 K recorded for *μ*_0_*H* = 0.1 T. It is clearly visible that 1/*χ*(*T*) curve has a distinctly nonlinear character. This phenomenon is a common feature of samarium containing compounds^19,35–37^, which origins from a relatively low energy of the excited state *J* = 7/2 above the ground state *J* = 5/2 for Sm^3+^ ion. In consequence the inverse susceptibility curve cannot be well described by a simple Curie–Weiss (CW) law, which should be substituted by the following formula^38^:

1 $$\chi \left( T \right) = \frac{{N_{A} \sum\nolimits_{J} {\left[ { \alpha_{J} + \frac{{g^{2} J\left( {J + 1} \right)\mu_{B} }}{{3k_{B} \left( {T - \theta_{CW} } \right)}}} \right]\left( {2J + 1} \right)e^{ - \Delta J/T} } }}{{\sum\nolimits_{J} {\left( {2J + 1} \right)e^{ - \Delta J/T} } }},$$

where *μ*_*B*_*, N*_*A*_, *θ*_*CW*_, and *k*_*B*_ are the Bohr magneton, the Avogadro number, the paramagnetic CW temperature, and the Boltzmann constant, respectively. The energy difference between the ground and excited multiplet is equal Δ*J* = 1494.9 K^37^ and the Van Vleck constant is represented by *α*_*J*_. Regarding high temperature region, the above equation can be rewritten as a formula:

2 $$\chi \left(T\right)=\left(\frac{{C}_\frac{5}{2}}{T-{\theta }_{\mathrm{CW}}}\right)+ {\alpha }_{J}+\left(\frac{{C}_\frac{7}{2}}{T}\right)\cdot {e}^{\frac{-\Delta }{T}}$$

which has four fitting parameters: *θ*_CW_, *α*_J_, the Curie constant for ^6^H_5/2_ state (C_5/2_) and ^6^H_7/2_ state (C_7/2_). The calculated values of these parameters, which are listed in Table 2, may be useful to estimate the effective magnetic moment applying the expression:

3 $${\mu }_{\mathrm{eff}}={\left(\frac{3C{\mathrm{k}}_{\mathrm{B}}}{{\upmu }_{\mathrm{B}}^{2}{\mathrm{N}}_{A}}\right)}^{1/2}$$

which leads to *μ*_eff_ equal 0.71 μ_B_ and 2.94 μ_B_ for ^6^H_5/2_ state and ^6^H_7/2_ state respectively. These experimental results are close to the theoretical data for Sm^3+^ (*μ*_theo_(^6^H_5/2_) = 0.84 μ_B_ and *μ*_theo_(^6^H_7/2_) = 2.58 μ_B_) calculated from Hund’s rule^39^, suggesting a trivalent nature of samarium in Sm_2_Au_1.1_Ge_2.9_. Moreover, the fit to the experimental data yields the positive value of *θ*_CW_ suggesting a ferromagnetic ground state.

**Table 2:** Selected physical parameters value for Sm_2_Au_1.1_Ge_2.9_.

Parameter	Sm_2_Au_1.1_Ge_2.9_
*T*_*irr*_(0) (K)	21.4
*T*_f_(39 Hz) (K)	21.9
δ*T*_f_ (-)	0.019
*E*_a_/k_B_ (K)	58.4(4)
*T*_0_ (K)	18.5(3)
*zυ*’ (-)	10.2(4)
*S* (emu/g)	2.32(4) × 10^–4^
*β* (-)	0.66(2)
ξ(2500 s, 10 K) (Å)	8733(6)
*θ*_CW_ (K)	21.89(2)
*μ*_eff_ (*μ*_B_)	0.71 (C_5/2_) / 2.94 (C_7/2_)
*ϕ*	1.1

**Fig. 6:**
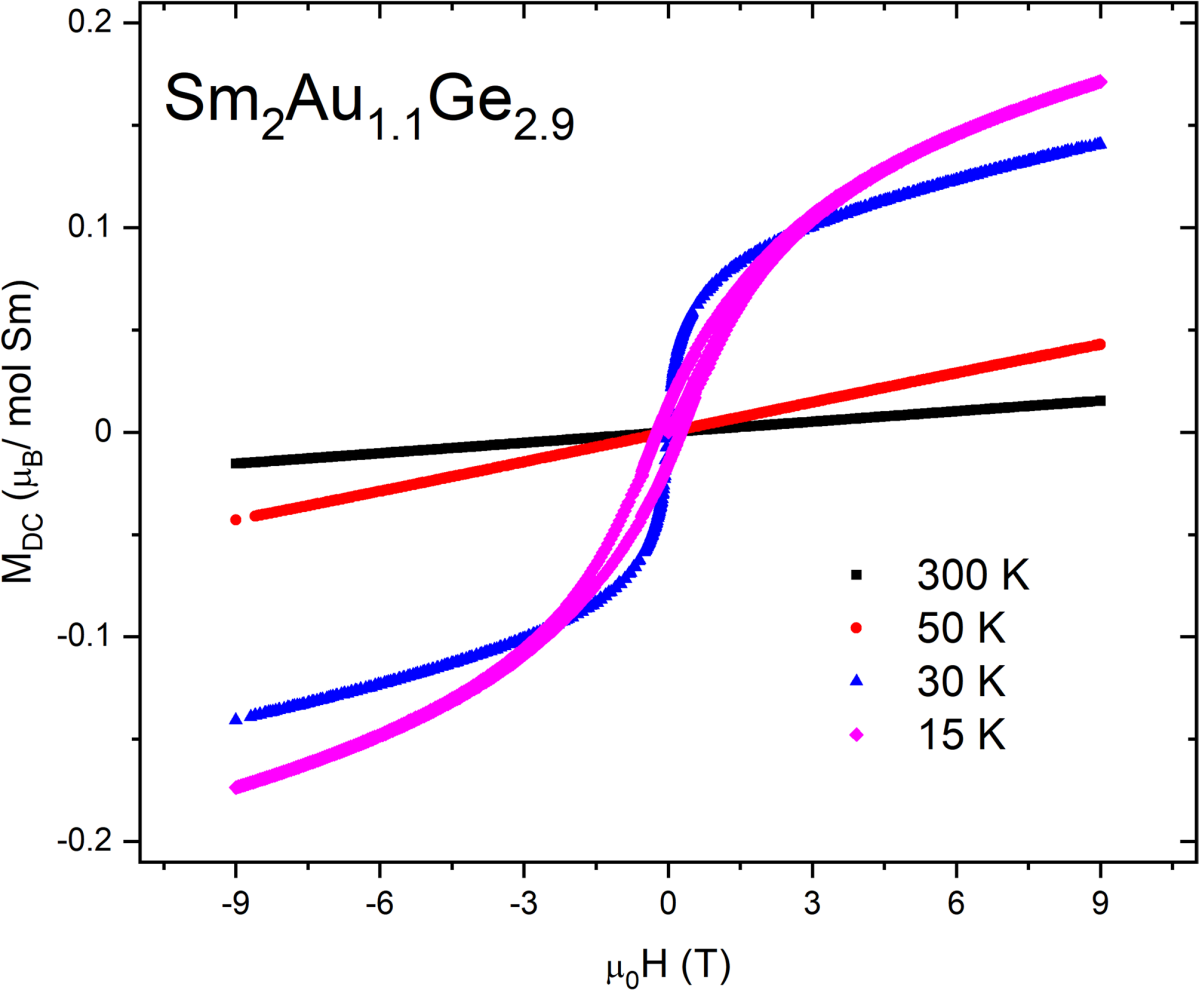
The isothermal magnetization as a function of an applied magnetic field at different temperatures for Sm_2_Au_1.1_Ge_2.9_.

The exact nature of magnetism in the described intermetallic compound was investigated by the *χ*(*T*) measurements in a low temperature region both in ZFC and FC mode for different applied magnetic fields. The gathered results are depicted in the inset (a) of Fig. 5, which shows an irreversibility between ZFC and FC curves near *T* = 20 K. The ZFC branch has evidence maximum at the thermal magnetic irreversibility temperature (*T*_irr_), and below this point the magnetic susceptibility continuously decreases with a temperature drop. Meanwhile the FC line is increasing with a decreasing temperature, which resembles rather ferromagnetic behavior instead reaching almost constant value expected for canonical spin glasses^40^. Moreover, the maximum value of *χ*_ZFC_ is about one order of magnitude larger than observed in a simple cluster glass Sm_2_PdGe_3_^19^, which may be a result of ferromagnetic clusters formation. These phenomena are characteristic features of a class of materials known as ferromagnetic spin glasses, which is represented by some members of *RE*_2_*TrX*_3_ family e.g. U_2_IrSi_3_^41^_,_ Eu_2_AuGe_3_^42^ and Ho_2_PtSi_3_^43^. Furthermore, the mentioned bifurcation tends to shift to lower temperatures with an increasing value of an applied magnetic field, which is clearly visible in the inset (b) of Fig. 6. The irreversibility temperature for each of applied magnetic fields was determined as a *dχ*_ZFC_ *(T*)/*dT* = 0 and for μ_0_*H* = 0.01 T its value is equal *T*_irr_ = 21.3 K. Cluster glasses usually obey de Almeida–Thouless dependence, which describes the dependence between *T*_irr_ and an applied magnetic field *H* and may be expressed by an equation^44^:

4 $$T_{irr} \left( {\mu_{0} H} \right) = T_{irr} \left( 0 \right)\left( {1 \, - A\mu_{0} H^{\kappa } } \right),$$

where *T*_irr_(0) ≈ 21.4 K is an irreversibility temperature in an absence of the external magnetic field and *A* is a constant. Following a mean field approximation for spin glasses *κ* = 2/3, while cluster glasses are characterized by a lower value of this parameter^45^. In the studied case the *T*_irr_(μ_0_*H*) plot is almost linear, which is another evidence of ferromagnetic clusters formation^2^. The occurrence of a glassy-like state is usually in line with an existence of a magnetic frustration, which may be described by an empirical criterion proposed by Ramirez: *ϕ* =|*θ*_cw_|/*T*_irr_^30^. For Sm_2_Au_1.1_Ge_2.9_ this parameter is close to 1, which is rather expected for compounds with long range ordering than for spin glass—like materials. However, this phenomenon was earlier observed for other ferromagnetic cluster glasses e.g. PrRhSn_3_^46^ and probably suggests a tendency to a magnetic ordering at the ground state. The further investigation of magnetic properties of Sm_2_Au_1.1_Ge_2.9_ included the isothermal magnetization measurements carried out at different temperatures.

Figure 6 presents the magnetization dependence *M*_DC_(μ₀*H*) measured across a temperature range of 15 K to 300 K. At higher temperatures, the *M*_DC_(μ₀*H*) curves exhibit an approximately linear behavior, consistent with the expected response of paramagnetic materials in the Curie–Weiss regime. Notably, the magnetization curve at *T* = 30 K does not show saturation, even under high magnetic fields, and displays an “S”-shaped profile characteristic of a Brillouin-like response. Such behavior has been previously reported for other members of the *RE₂TrX₃* family^11,14,18,19^, and is indicative of spin glass–like magnetic behavior. At T = 15 K, the emergence of a small hysteresis loop was observed, which may be attributed to the presence of a secondary magnetic impurity phase. However, similar phenomena have also been reported for other compounds within the *RE₂TrGe₃* series^27,47^, suggesting this feature may be intrinsic to the structural family.

**Fig. 7:**
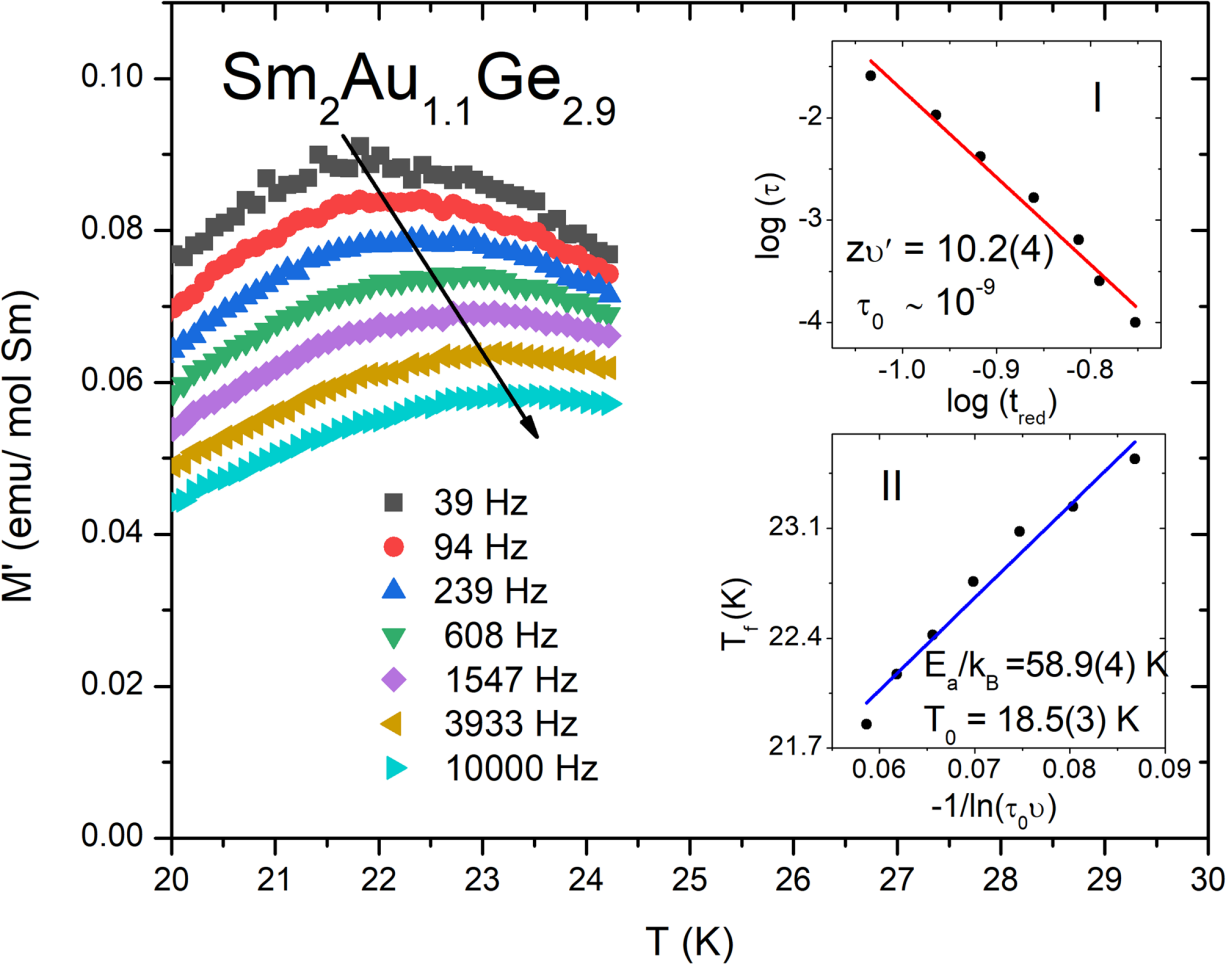
Temperature dependence of the real part of the AC magnetization *M*’(*T*) for Sm_2_Au_1.1_Ge_2.9_. The inset (I) shows ln(*τ*) plotted as a function of ln(*t*_red_) with the solid red line, which represents the fit to the power-law divergence. The inset (II) shows plot of the freezing temperature (*T*_f_) versus 1/ln(*τ*_0_*υ*) with a Vogel-Fulcher law fit (blue solid line).

The appropriate classification of the magnetic transition at *T*_irr_ requires also AC magnetization studies, which results are depicted in Fig. 7. The clearly visible peak at *M*’(*T*) appears close to *T*_irr_ and strongly depends on the applied AC frequency, which is a one of characteristic features of spin glass-like materials^1^. The increase of *υ* cause a shift of the observed maximum to higher temperatures and its value at the lowest measured frequency is usually employed to determine a freezing temperature *T*_f_ = 21.9 K. The relative shift of this parameter may be described by a criterion proposed by Mydosh^4^:

5 $$\updelta {T}_{f} =\frac{\Delta {T}_{f}}{{T}_{f}\Delta \mathrm{log}\upsilon }$$

The calculated value of δ*T*_*f*_ = 0.019 is an order of magnitude larger than expected for canonical spin glasses (~ 10^–3^), although it is in range typical for cluster glass materials for example CeCu_4_Mn_0.9_Al_0.1_—δ*T*_*f*_ = 0.0177(1)^48^, Er_2_NiSi_3_—δ*T*_*f*_ = 0.02^11^, and Sm_2_PdGe_3_—δ*T*_*f*_ = 0.016^19^. The AC measurements data can be further analyzed applying a dynamical scaling theory, which may be expressed by a power law dependence^49^:

6 $$\tau = \tau_{0} \left( {\frac{{T_{f} - T_{SG} }}{{T_{SG} }}} \right)^{{ - zv^{\prime } }}$$

where *τ* ~ 1/*υ* is the relaxation time associated with a measurement frequency *υ* and *zυ*’ is a dynamic critical exponent, which is related to a correlation length (ξ = (*T*_*f*_ /*T*_SG_ − 1)^− *υ*’^, *τ* ∼ ξ^*z*^)^11^. The parameter *τ*_0_ is a characteristic flipping time of the magnetic entities and vary from *τ*_0_ = 10^–7^ s for cluster glasses to *τ*_0_ = 10^–13^ s for canonical spin glasses^1^. The spin glass temperature (*T*_SG_) is defined as a temperature in the static limit (*υ* → 0), however for simplicity is usually equated with *T*_irr_*.* The fit of the above formula to depicted in the inset I of Fig. 7 log(*τ*) versus log(*t*_*red*_) plot (*t*_*red*_ is a reduced temperature and may be established as *t*_*red*_ = (*T*_*f*_—*T*_SG_)/*T*_SG_) lead to values of *zυ*’ and *τ*_0_ estimated as 10.2(4) and 10^–9^ s respectively, which indicate the cluster glass transition in Sm_2_Au_1.1_Ge_2.9_. Another possibility to explain the spin glass-like phenomenon is regarding *T*_f_ as a temperature of a thermal activation of interactions between magnetic spins, which may be denoted by an empirical Vogel-Fulcher (VF) law^50^:

7 $$\uptau = {\uptau }_{0} exp\left(\frac{{E}_{a}}{{\mathrm{k}}_{\mathrm{B}}\left({T}_{f}-{T}_{0}\right)}\right),$$

where *E*_a_ is an activation energy and *T*_0_ means the measure of interaction strength between magnetic spins, which is known as a VF temperature. This equation can be also referred as a dependence between *T*_f_ and *υ*:

8 $${T}_{f}={T}_{0}-\frac{{E}_{\mathrm{a}}}{{\mathrm{k}}_{\mathrm{B}}}\frac{1}{\mathrm{ln}\left({\tau }_{0}\upsilon \right)},$$

which is useful to determine *E*_a_ and *T*_0_ from a linear fit to *T*_f_ vs. − 1/ln(*τ*_0_*υ*) plot (the inset II of Fig. 7). Obtained values of these parameters are gathered in Table 2. The *E*_a_k_B_/*T*_0_ ratio is much greater than one, which suggests a weak coupling between magnetic entities and confirm the hypothesis about the cluster glass formation^49^. Finally, this assumption is in accordance with a Tholence criterion^50^: δ*T*_Th_ = (*T*_f_ – *T*_0_)/ *T*_f_ ≈ 0.16, which gives a similar result to previously reported *RE*_2_*TrX*_3_ compounds e.g. δ*T*_Th_ = 0.12 for Nd_2_PtGe_3_^15^, δ*T*_Th_ = 0.32 for Ho_2_Pd_1.3_Ge_2.7_^29^ and δ*T*_Th_ = 0.10(2) for Ce_2_PdGe_3_^51^.

**Fig. 8:**
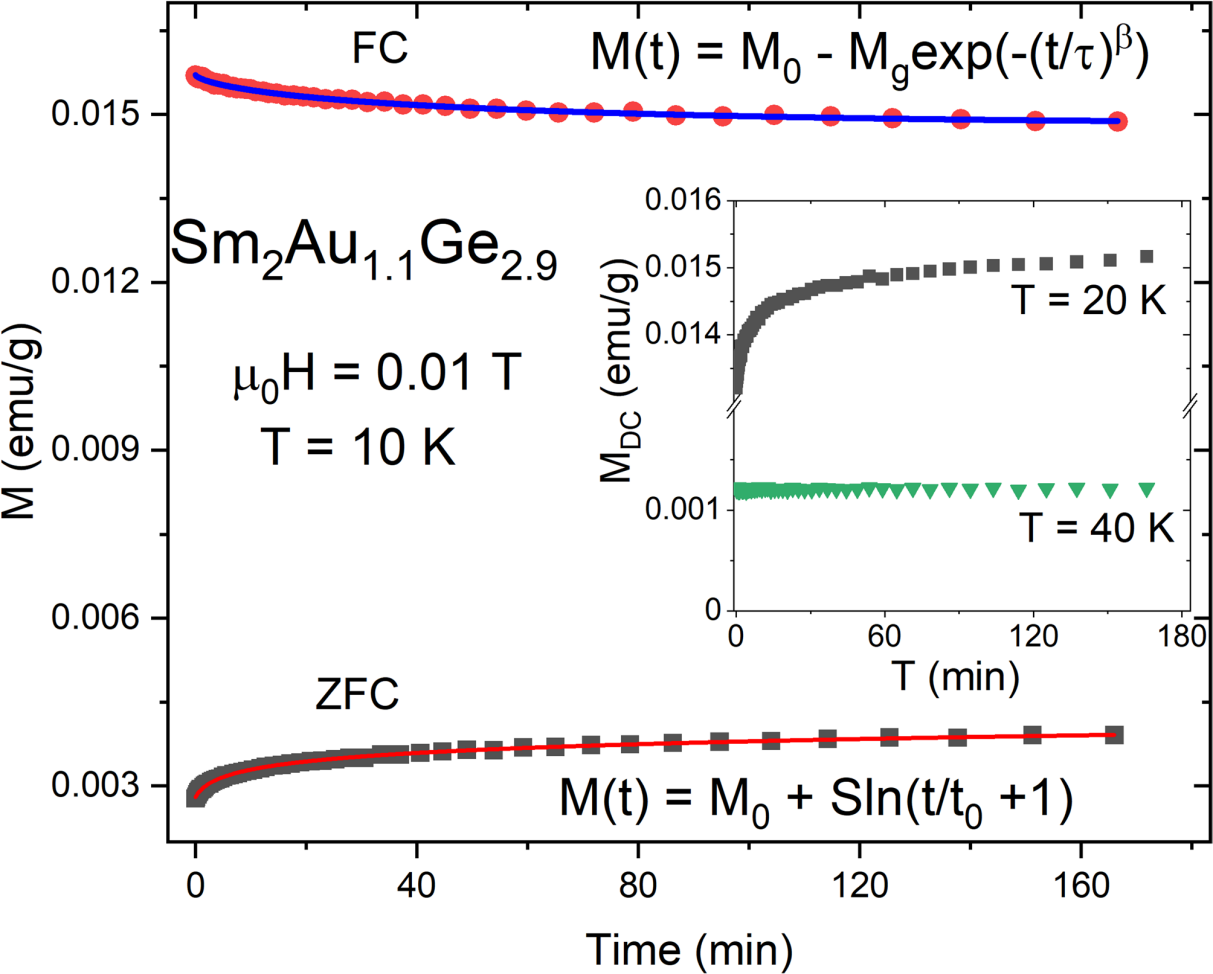
Time dependent remnant magnetization for Sm_2_Au_1.1_Ge_2.9_ measured in ZFC and FC mode at *T* = 10 K. Red line represents a fit to the equation *M*(*t*) = *M*(0) + *S*ln(*t*/*t*_0_ + 1) and a blue one exhibits a fit of the stretched exponential law: *M* = *M*_0_ – *M*_g_exp(-(*t*/*τ*)^*β*^). The inset exhibit time dependent remnant magnetization behavior for Sm_2_Au_1.1_Ge_2.9_ measured at ZFC mode at different temperatures.

In order to avoid any ambiguity in interpretation of the spin-glass phenomena, the time dependent DC magnetization for Sm_2_Au_1.1_Ge_2.9_ was measured in ZFC as well as in FC mode, which were described in details in previously published articles^11,43,52^. The collected data are presented in Fig. 8, which appears that the studied compound exhibits a time evolution of magnetization known as an aging effect^1,2^. This occurrence may be explained by a nonequilibrium dynamics below *T*_f_ which arise from a magnetization reorientation. The magnetic relaxation behavior in spin glass—like compounds obeys a stretched exponential law expressed by a function^49^:

9 $$M\left( t \right) = M_{0} \pm M_{g} \exp \left( { - \left( {t/\tau } \right)^{\beta } } \right),$$

where *M*_0_ corresponds to an initial magnetization defined as magnetization at *t* = 0, *M*_g_ describes a glassy component of the magnetization and *τ* is a characteristic relaxation time. The last of fitting parameters – *β* signifies a stretching exponent, which for a different glassy systems lies in the range 0 < *β* < 1, where *β* = 0 imply a lack of relaxation^53^. The alternative method to describe the relaxation phenomenon is using a logarithmic dependence^52^:

10 $$\left( t \right) = M_{0} \pm S\ln \left( {t/t_{0} + 1} \right),$$

where *S* is the magnetic viscosity. The reference time *t*_0_ has only limited physical relevance and is usually orders of magnitude larger than *τ*_0_^50^. All of these parameters depend on measuring conditions, especially from a temperature and an applied magnetic field. The values of fitting parameters estimated using both of mentioned above equations are listed in Table 2 and correspond to results reported for other cluster glasses from *RE*_2_*TrX*_3_series^11,14,15,52,54^,. The mismatch between the initial magnetization calculated from the first equation (*M*_0_ = 0.01479(9) emu/g) and the second one (*M*_0_ = 0.0028(2) emu/g) is a consequence of a magnetic memory effect, which is further discussed. The inset of Fig. 8 shows a time dependence of magnetization at different temperatures. It is obvious that the ageing effect occurs only below *T*_*f*_ and disappears at higher temperatures. This phenomenon confirms our assumption about spin glass—like transition in Sm_2_Au_1.1_Ge_2.9_.

**Fig. 9:**
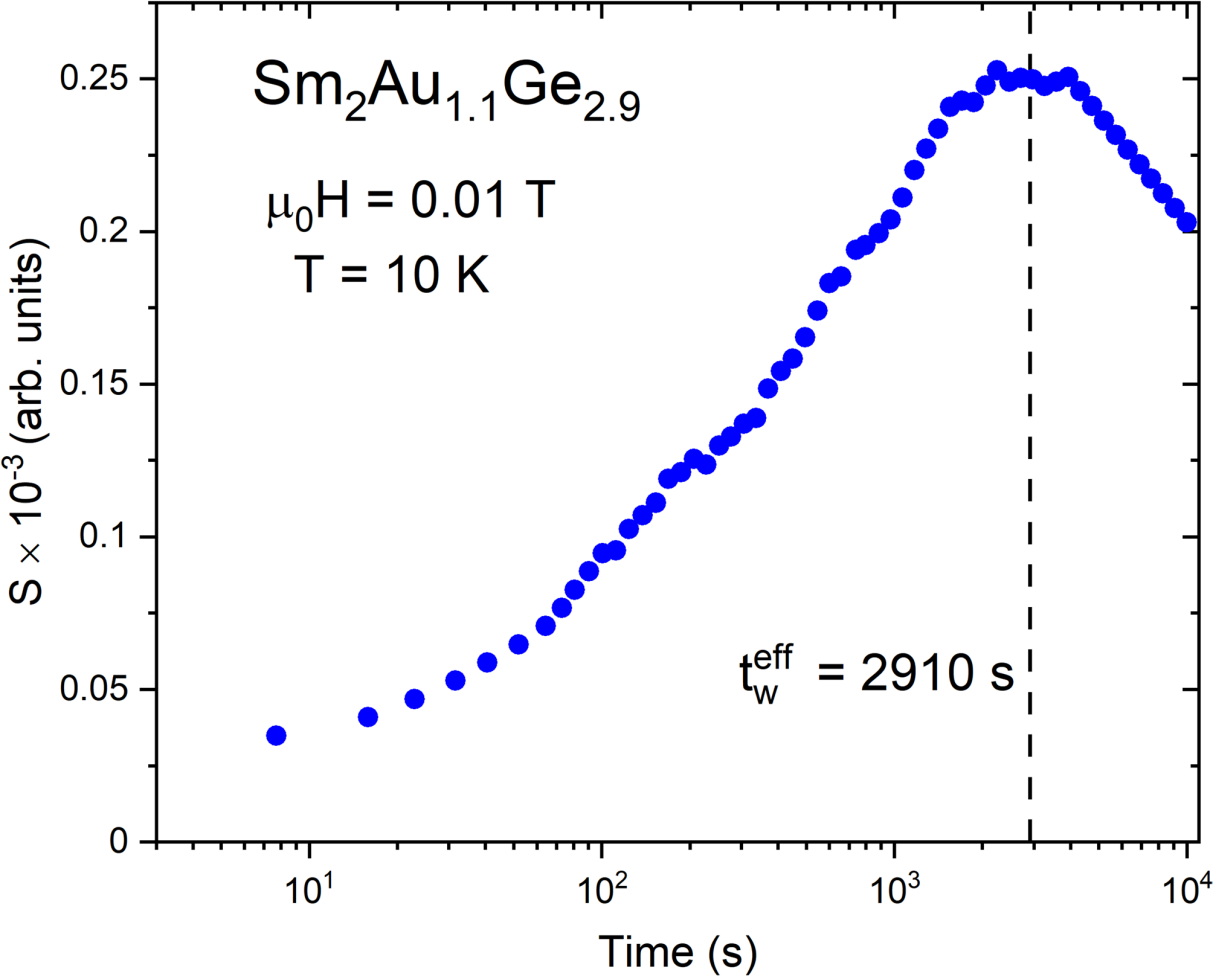
The relaxation rate S(t) for Sm_2_Au_1.1_Ge_2.9_ (discussed in the text) at T = 10 K and in a magnetic field of 0.01 T after waiting for *t*_*w*_ = 2500 s in zero applied magnetic field.

The non-equilibrium dynamics in spin glass—like materials can be also observed in a time evolution of the magnetic viscosity^55^, which is shown in Fig. 9. The time dependent magnetic viscosity can be described by the response function proposed by Joh^56^:

11 $$S\left( t \right) = \frac{{d\left[ { - M_{FC} \left( t \right)/H} \right]}}{d\ln t}$$

where *M*_*FC*_ means the thermoremanent magnetization in a function of time after switching off an external magnetic field *H*. The curve *S(t)* for T = 10 K presented in Fig. 9 exhibits a maximum, which can be employed to estimate the effective waiting time ${t}_{w}^{eff}$. This parameter is usually larger than *t*_*w*_ resulting from the cooling protocol^55^. The average cluster size is usually described by a quantity called the coherence length (ξ), which can be calculated by knowing the parameters ${t}_{w}^{eff}$ and *t*_*w*_. For this purpose, should be used the formula^56^:

12 $${E}_{Z}={k}_{B}T\left({ ln{t}_{w}-lnt}_{w}^{eff}\right)$$

where *E*_*z*_ is a Zeeman energy, which can be interpreted as a magnetic energy associated with a change in magnetic field. The Zeeman energy is directly correlated with a volume within which the spins (*N*_*s*_) are effectively locked together during barrier hopping by an equation^57^:

13 $$E_{Z} = N_{s} \chi_{FC} H^{2} ,$$

where *χ*_*FC*_ is the spin glass susceptibility per spin measured in the FC conditions. The parameter *N*_*s*_ stands for the number of correlated spin units that flip together within a volume ξ^3^, thus the correlation length can be described as:

14 $$\upxi =\sqrt[3]{{N}_{s}}$$

In case of Sm_2_Au_1.1_Ge_2.9_ the calculated value of ξ was equal 8733(6) Å, which is about an order of magnitude larger than typical values for spin glasses^57,58^. This result is in a good agreement with the hypothesis about a cluster glass transition in the investigated sample.

**Fig. 10:**
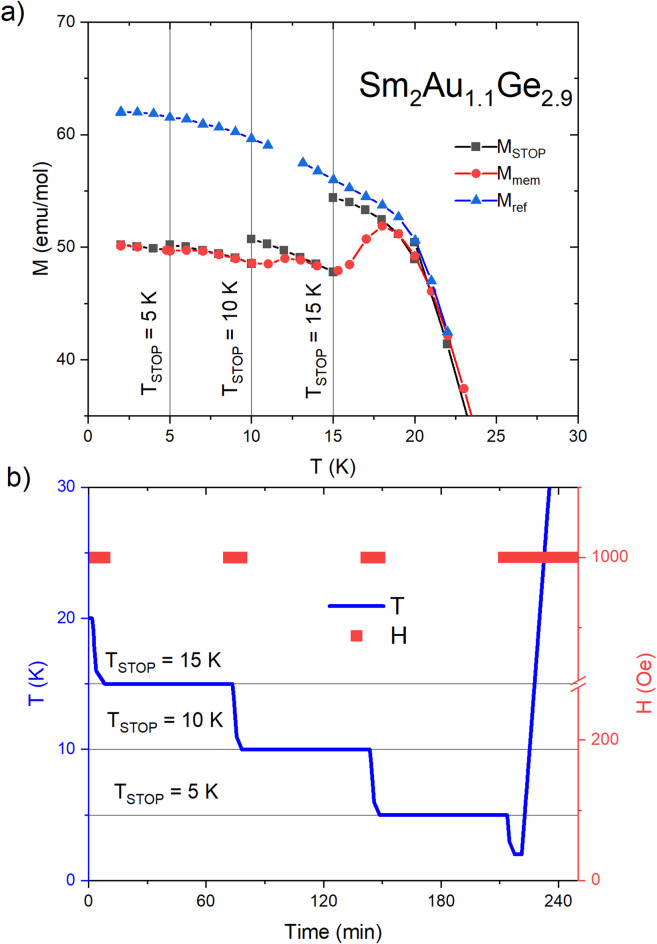
(**a**) Memory effect of Sm_2_Au_1.1_Ge_2.9_ measured in FC protocol The *M*_stop_ denotes the results obtained in the cooling process, the *M*_mem_ denotes the results obtained in the heating process and the *M*_ref_ denotes the reference results obtained in the standard FC process. (**b**) The FC protocol used to obtain memory effect.

Another intriguing property of spin glass—like materials is a magnetic memory effect, which is also a result of non-equilibrium dynamics occurring at different glassy systems. This phenomenon may be observed employing a procedure proposed by Sun et al.^59^. The sample of Sm_2_Au_1.1_Ge_2.9_ was cooled down from a paramagnetic region to the lowest attainable temperature (*T* = 2 K for PPMS device) in the presence of an external magnetic field μ_0_*H* = 0.01 T. The cooling process was stopped at chosen temperatures below *T*_f_ (*T*_stop_ = 15 K, 10 K and 5 K) in which the sample was maintained at zero field conditions for a waiting time *t*_w_ = 1 h. Then the same magnetic field was reapplied, and the cooling was resumed. After attaining *T* = 2 K, the *M*(*T*) dependence was measured during heating with the same applied magnetic field. Figure 10 presents data collected employing the mentioned above procedure. This plot clearly indicates that the magnetic memory effect can be found in the sample investigated. The observed peculiarities of *M*_mem_ curve are similar to those revealed for various spin glass-like materials^11,43,52,60^ and are in line with a presumption about the spin glass—like state formation.

### Specific heat

**Fig. 11:**
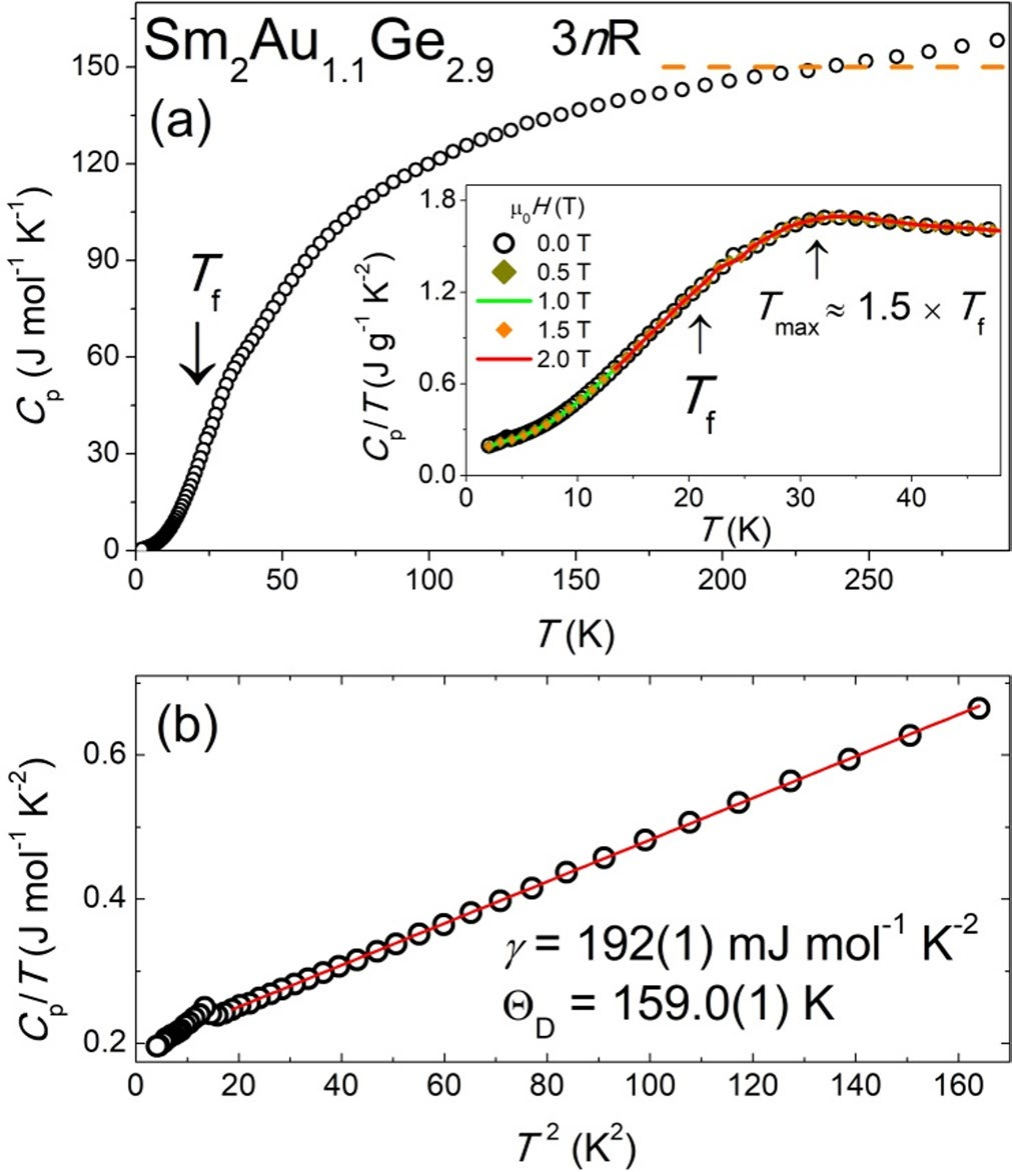
Specific heat (*C*_p_) of Sm_2_Au_1.1_Ge_2.9_. (**a**) Temperature dependence of (*C*_p_) vs. *T*. Dashed line represents Dulong-Petit limit. The insets shore presents *T* in the low temperature range measured in zero and finite magnetic field *μ*_0_*H*. *T*_f_—freezing temperature from magnetic measurements, *T*_max_—maximum in temperature dependence of specific heat. (**b**) Plot of *C*_p_/*T* vs. *T*^2^ at low temperatures. The solid line represents fit to Debye model (Eq. 15).

The heat capacity measurements were carried out in order to confirm the volume character of observed in Sm_2_Au_1.1_Ge_2.9_ magnetic transitions. Figure 11a shows the temperature dependence of the specific heat gathered in the absence of an external magnetic field. It should be noted that the *C*_p_(*T*) plot reaches saturation at room temperature slightly above the value calculated from the Dulong-Petit limit: 3*n*R ≈ 150 J mol^−1^ K^−1^, where *n* is the number of atoms per formula unit (*n* = 6) and R is the gas constant (R = 8.314 J mol^−1^ K^−1^). This behavior may originate from an electron excitation of Sm ions or be related to the anharmonicity effect resulting from structural disorder^61^.

The *C*_p_(*T*) dependence does not have a sharp maximum typical for materials with long-range magnetic ordering. The absence of such an anomaly is typical for spin glass-type systems^1^. At temperatures higher than *T*_*f*_ determined from magnetic measurements, a broad shoulder is seen. In addition, the magnetic field has little effect on the observed anomaly. Furthermore, the inset of Fig. 11a reveals a small kink around 30 K, which is most likely associated with trace amounts of secondary phases, such as Sm₂Au or Sm₂AuGe₆.

At *T* ≈ 4 K, a small anomaly was observed, which most likely comes from an impurity phase. Taking into account the results of pXRD and SEM/EDS measurements, it can be assumed that this phase transition is related to the presence of Sm_2_AuGe_6_.

The range of lowest temperatures (Fig. 11b) can be described by the simplified Debye equation^62^:

15 $$C_{p} = \gamma T \, + \, 12/5\pi^{4} nR(T/\Theta_{D} )^{3} + \, \left( {AT \, + \, BT^{2} } \right),$$

where *γ* is electronic specific heat coefficient and Θ_D_ is the Debye temperature. Fitting carried out in the range from 5 to 12 K yielded the following values of the parameters *γ* = 192(1) mJ mol^−1^ K^−1^ and Θ_D_ = 159.0(1) K. The value of the *γ* is significantly higher (by two orders of magnitude) than for simple metals and their alloys. An increase in *γ* value can be related to various effects such as heavy-fermion-type behavior, spin fluctuations, crystal electric field, disorder, etc. The values obtained are typical for this type of material^51,63^. Using the formula^62^:

16 $$\gamma = k_{B}^{2} p^{4} N\left( {E_{F} } \right)/3$$

the electronic density of states *N*(*E*_F_) at the Fermi level was also determined and is equal to 81 states/(eV atom). Measurements of specific heat and their analysis confirm that there are no signs of long-range magnetic ordering. The absence of a clear anomaly, along with the material’s low sensitivity to changes in the magnetic field and a large *γ* coefficient, suggests that this material behaves like a spin-glass.

## Conclusions

The intermetallic compound Sm_2_Au_1.1_Ge_2.9_ is the member of the *RE*_2_AuGe_3_ family which crystallizes in hexagonal structure (space group *P*6/*mmm*). This material was synthetized using an arc melting technique and can be obtained in single phase only by deliberately tweaking the nominal stoichiometry. The pXRD investigations indicated that the compound studied crystallize in a disordered variant of the AlB_2_ derived structure, which promotes a spin glass-like state formation. Lattice parameters, which were calculated using the Rietveld refinement are equal *a* = 4.2495(1) Å and *c* = 4.1300(1) Å. Magnetic susceptibility and specific heat measurements reveal that the sample of Sm_2_Au_1.1_Ge_2.9_ exhibits a spin glass—like ordering. It can be observed at *T*_f_ (39 Hz) = 21.9 K the transition from paramagnet to ferromagnetic cluster glass, which is confirmed both by the analysis of AC and DC susceptibility measurements. The compound exhibits the magnetic memory effect. The presented results pave the way for future research in *RE*_2_AuGe_3_ family, which is a promising candidate for investigations of novel materials with application potential in magnetic memory devices. Further research on this intermetallic compound may include measurements of its electrical properties and magnetocaloric studies.

## Data Availability

The datasets generated during and/or analysed during the current study are available from the corresponding author on reasonable request.
